# The role of transcriptomic biomarkers of endometrial receptivity in personalized embryo transfer for patients with repeated implantation failure

**DOI:** 10.1186/s12967-021-02837-y

**Published:** 2021-04-28

**Authors:** Aihua He, Yangyun Zou, Cheng Wan, Jing Zhao, Qiong Zhang, Zhongyuan Yao, Fen Tian, Hong Wu, Xi Huang, Jing Fu, Chunxu Hu, Yue Sun, Lan Xiao, Tianli Yang, Zhaojuan Hou, Xin Dong, Sijia Lu, Yanping Li

**Affiliations:** 1 Department of Reproductive Medicine, Xiangya Hospital, Central South University, 87 Xiangya Road, Changsha, 410000 Hunan China; 2 Clinical Research Center for Women’s Reproductive Health in Hunan Province, Changsha, 410000 Hunan China; 3 Department of Clinical Research, Yikon Genomics Company, Ltd., #301, Building A3, No. 218, Xinghu Street, Suzhou, 215123 Jiangsu China; 4 Department of ENT, Xiangya Hospital, Central South University, Changsha, 410000 Hunan China; 5 Key Laboratory of Otolaryngology in Hunan Province, Changsha, 410000 Hunan China

**Keywords:** Endometrial receptivity, Window of implantation, Biomarkers, RNA-Seq, Repeated implantation failure, Personalized embryo transfer

## Abstract

**Background:**

Window of implantation (WOI) displacement is one of the endometrial origins of embryo implantation failure, especially repeated implantation failure (RIF). An accurate prediction tool for endometrial receptivity (ER) is extraordinarily needed to precisely guide successful embryo implantation. We aimed to establish an RNA-Seq-based endometrial receptivity test (rsERT) tool using transcriptomic biomarkers and to evaluate the benefit of personalized embryo transfer (pET) guided by this tool in patients with RIF.

**Methods:**

This was a two-phase strategy comprising tool establishment with retrospective data and benefit evaluation with a prospective, nonrandomized controlled trial. In the first phase, rsERT was established by sequencing and analyzing the RNA of endometrial tissues from 50 IVF patients with normal WOI timing. In the second phase, 142 patients with RIF were recruited and grouped by patient self-selection (experimental group, n = 56; control group, n = 86). pET guided by rsERT was performed in the experimental group and conventional ET in the control group.

**Results:**

The rsERT, comprising 175 biomarker genes, showed an average accuracy of 98.4% by using tenfold cross-validation. The intrauterine pregnancy rate (IPR) of the experimental group (50.0%) was significantly improved compared to that (23.7%) of the control group (RR, 2.107; 95% CI 1.159 to 3.830; *P* = 0.017) when transferring day-3 embryos. Although not significantly different, the IPR of the experimental group (63.6%) was still 20 percentage points higher than that (40.7%) of the control group (RR, 1.562; 95% CI 0.898 to 2.718; *P* = 0.111) when transferring blastocysts.

**Conclusions:**

The rsERT was developed to accurately predict the WOI period and significantly improve the pregnancy outcomes of patients with RIF, indicating the clinical potential of rsERT-guided pET.

*Trial registration* Chinese Clinical Trial Registry: ChiCTR-DDD-17013375. Registered 14 November 2017, http://www.chictr.org.cn/index.aspx

**Supplementary Information:**

The online version contains supplementary material available at 10.1186/s12967-021-02837-y.

## Background

A successful pregnancy depends on a successful human embryo implantation, which requires a receptive endometrium, a normal and functional embryo at the blastocyst developmental stage and a synchronized dialog between the maternal and embryonic tissues [1]. Inadequate endometrial receptivity (ER), defined as the ability of the endometrium to accept and accommodate a nascent embryo, is responsible for approximately two-thirds of implantation failures [2]. This period of receptivity is known as the “window of implantation” (WOI) [3], which usually occurs on days 19–24 of the menstrual cycle [4], on day 7 after the LH surge (LH + 7) in the natural cycle or on day 5 after progesterone administration (P + 5) in the artificial cycle [2, 5]. Traditionally, the WOI was thought to be quite wide, but the optimal window has been shown to be much smaller, possibly lasting for only 2 days [6]. Delayed implantation at the extremes of the endometrial window can result in poor pregnancy outcomes [7]. Therefore, an objective and accurate determination of the optimal WOI is crucial for improving the outcomes of pregnancy facilitated by assisted reproductive technology (ART).

The length of the WOI is not consistent among all women, and some present with WOI displacement, which can delay, advance or narrow the WOI [8]. This can contribute to embryo-endometrial asynchrony, which usually results in implantation failure or even repeated implantation failure (RIF) [9], defined by some as a minimum of two IVF or frozen cycle failures in which at least four morphologically high-quality cleavage-stage embryos or two high-quality blastocysts were transferred [10]. In IVF-embryo transfer (ET), the incidence of RIF is as high as 5–10% [11], and approximately 60% of RIF can be attributed to abnormal maternal ER, which presents as a displacement and/or pathological disruption of the WOI [12]. Displacement of WOI is present in 1 of 4 patients with RIF [13]. Thus, the accurate identification of the optimal WOI in patients with RIF by an effective diagnostic tool and subsequent personalized embryo transfer (pET) to restore synchronicity of embryonic and endometrial development can allow for successful embryo implantation [14].

The study of ER dates back to the 1950s, when Noyes et al. established classical histological criteria for the evaluation of ER by analyzing endometrial staging and receptivity status [15]. Many studies have sought to define a healthy WOI by various ER markers through ultrasonography [16–20], morphology [21], and molecular biology [22–25]. However, the objectivity, accuracy, reproducibility and functional relevance of these studies have been questioned [26–28].

As new high-throughput “omics” studies have emerged, the endometrial transcriptome has provided a deeper understanding of ER [29], and the feasibility of a molecular diagnostic tool that can identify a receptive endometrium based on a specific transcriptomic signature in different stages of the endometrial cycle has been demonstrated [30–32]. For example, an endometrial receptivity array (ERA) based on microarray technology coupled to a computational predictor was able to identify the WOI by predicting the receptivity status of endometrial biopsy samples objectively and accurately. The ERA contains 238 genes that were screened from the differential gene expression profiles of the prereceptive versus receptive status [33]. The ERA is more accurate than histological methods, and the results have been shown to be reproducible in the same patients 29–40 months after the first test [34]. Moreover, these studies have demonstrated the clinical value of ERA in patients with RIF for guiding pET [35]. A recent multicenter randomized clinical trial indicated the potential of the ERA test in the diagnosis of endometrial factors in the work-ups of infertile couples at their first appointments [36]. Before performing pET, infertile patients underwent one or two endometrial biopsies for the ERA test to accurately determine the receptive period. Their pregnancy, implantation and cumulative live birth rates were statistically significantly improved by pET guided by the ERA test. This provides a basis for further development of ER molecular diagnostic tools for reproductive medicine clinics through endometrial transcriptome research.

RNA-Seq is a new-generation high-throughput sequencing technique used in transcriptomics research. Compared with conventional microarrays, RNA-Seq has the benefits of ultra-high sensitivity, dynamic range, more accurate quantification, and whole-transcriptome analysis, which would allow identification of differentially expressed genes (DEGs) for ER from an unrestricted range of genes [37]. ER diagnostic methods can be further improved by transcriptomic analysis of ER using RNA-Seq.

To improve molecular diagnostic tools for ER, we improved the experimental design and endometrial biopsy sampling time and combined them with a machine learning algorithm to construct a novel RNA-Seq-based endometrial receptivity test (rsERT) consisting of ER-specific marker genes and to investigate whether pET guided by rsERT can improve pregnancy outcomes in patients with RIF.

## Methods

### Study design and participants

This study was conducted at the Department for Reproductive Medicine at Xiangya Hospital in Changsha, Hunan, People's Republic of China. The study was approved by the Reproductive Medicine Ethics Committee of Xiangya Hospital and was registered in the Chinese Clinical Trial Registry (registration no. ChiCTR-DDD-17013375).

All patients were undergoing IVF between November 2017 and July 2019. This study describes two separate phases.

In the first phase, from November 2017 to December 2018, participants were recruited to identify DEGs among the prereceptive, receptive and postreceptive endometrium and to build the rsERT. To limit interference from confounding variables affecting ER, the inclusion criteria for IVF patients were as follows: 20–39 years of age; body mass index (BMI) = 18–25 kg/m^2^; patients with a history of a intrauterine pregnancy/pregnancies who underwent the first IVF cycle due to tubal factors alone; patients who undergoing the first IVF cycle due to male factors alone; a regular menstrual cycle length (25–35 days) with spontaneous ovulation; normal ovarian reserve (baseline FSH < 10 mIU/mL, anti-Mullerian hormone > 1.5 ng/ml, antral follicle count > 5); able to be followed up to assess the pregnancy outcome; and successful intrauterine pregnancy after the first ET. Intrauterine pregnancy was defined as the presence of a gestational sac with or without fetal heart activity in the uterine cavity as evaluated by ultrasound 4–5 weeks after ET. To establish the prediction tool, normal ER status was defined as successful intrauterine pregnancy.

In the second phase, from May 2018 to July 2019, participants were recruited to demonstrate the clinical impact of rsERT in patients with RIF. This study was designed as a prospective, nonrandomized concurrent controlled trial. No reliable data were available at the trial design to allow for an accurate sample size calculation. Therefore, based on the results of the pre-experiment, we used the assumption that the intrauterine pregnancy rate was 60% in the experimental group and 25% in the control group and considered a two-sided *P*-value to be deemed statistically significant at *P* < 0.05 and a power of 80%. Considering a 10% loss-to-follow-up rate, 33 subjects were required in each group. The calculations of sample size were conducted with PASS software (version 11.0). The inclusion criteria for patients with RIF were as follows: 20–39 years of age; BMI = 18–25 kg/m^2^; and a history of RIF, which was defined as failure to achieve a clinical pregnancy after the transfer of at least 4 morphologically high-quality cleavage-stage embryos or 2 high-quality blastocysts in a minimum of 2 fresh or frozen cycles. The criteria for good-quality embryos were as follows: (i) cleavage-stage embryos: ≥ 7 blastomeres and < 20% fragmentation on day 3 after fertilization [38, 39] and (ii) blastocysts: ≥ 3 BB on day 5 and day 6, graded based on the Gardner system [40]. After providing informed consent, patients with RIF who chose to receive the rsERT to predict and guide pET were included in the experimental group, and those who chose not to receive the rsERT and underwent conventional ET directly were included in the control group.

The following exclusion criteria were applied: endometrial diseases (including intrauterine adhesions, endometrial polyps, endometritis, endometrial tuberculosis, endometrial hyperplasia, and a thin endometrium); hydrosalpinx without proximal tubal ligation; submucous myomas, intramural hysteromyomas, or adenomyomas protruding towards the uterine cavity; endometriosis (stages III–IV); uterine malformations; and other medical or surgical comorbidities were identified by consulting medical records, physical examination, blood test, B-ultrasound and X-ray examination.

All patients were followed up to assess pregnancy outcomes, as follows: the grade and number of embryos transferred for all participants were recorded. Blood β-human chorionic gonadotropin (β-HCG) was measured 12 days after ET, and the intrauterine pregnancy and number of gestational sacs were assessed by ultrasound 28 days after transfer in β-HCG-positive patients. Subsequently, all patients diagnosed with an intrauterine pregnancy were followed up until delivery. The last follow-up date was May 2020.

### Endometrial biopsy, sample collection and processing

Written informed consent was obtained before sample collection. For patients included in the model construction phase, ultrasound was initiated from day 10 of the menstrual cycle preceding the IVF cycle to monitor ovulation. Blood LH levels were dynamically measured daily when the diameter of the dominant follicle was ≥ 14 mm. Patients continued to undergo daily ultrasound monitoring of ovulation until follicular discharge. Endometrial tissues were collected using an endometrial sampler (AiMu Medical Science & Technology Co.; Liaoning; China) on days 5, 7, and 9 (LH + 5, LH + 7, and LH + 9, respectively) after the LH surge (denoted as LH + 0).

For patients with RIF in the experimental group with a natural cycle, the timing of endometrial tissue sampling was the same as that in the modeling group above. For hormone replacement (HRT) cycles, estradiol administration was started on the third day of the menstrual cycle, and progesterone supplementation was started after at least 12 days of estrogen usage if the endometrium was > 7 mm and the endogenous P serum level was close to zero. The day of starting progesterone supplementation was considered P + 0, and endometrial tissues were collected on days 3, 5, and 7 after progesterone supplementation (i.e., on days P + 3, P + 5, and P + 7, respectively).

In all cases the sampling was performed as follows. The cervix was cleansed with saline before sampling. The tip of the endometrial sampler was placed into the uterine fundus, and 5–10 mm^3^ of endometrial tissues were aspirated into the sampler. The collected endometrial tissues were immediately placed into 1.5 mL RNA-later buffer (AM7020; Thermo Fisher Scientific, Waltham, MA, USA) for RNA stabilization, sealed, and cryopreserved at − 20 °C. Sequencing analysis was carried out within 7 days after sampling.

### RNA extraction, library construction and sequencing

Total RNA extraction was performed using the RNeasy Micro Kit (74004; Qiagen, Germantown, MD, USA) according to the instruction manual, followed by quantification with a Qubit HS RNA Kit (Q32855; Thermo Fisher Scientific, Waltham, MA, USA). Then, an RNA LabChip (Agilent Technologies, Santa Clara, CA, USA) was used in combination with an Agilent 2100 Bioanalyzer (Agilent Technologies, Santa Clara, CA, USA) for integrity and quality control of the extracted total RNA. Samples with an RNA integrity number (RIN) > 7 were considered eligible for subsequent tests.

RNA reverse transcription was conducted using the MALBAC® Platinum Single Cell RNA Amplification Kit (KT110700796; Yikon Genomics, Suzhou, Jiangsu, China) according to the instruction manual. Both positive and negative controls, consisting of 500 ng of high-quality human total RNA and ultrapure water, respectively, were included to ensure that the experiments were conducted properly. For this step, 1 µl purified cDNA was reasonably diluted for detection on the Agilent 2100 Bioanalyzer High Sensitivity DNA Chip (Agilent Technologies, Santa Clara, CA, USA) according to the instruction manual. cDNA with a size of 1000–10,000 bp met the quality control requirements.

Library construction was accomplished using a gene sequencing and library preparation kit (XY045; Yikon Genomics, Suzhou, Jiangsu, China) according to the instruction manual. After purification, the libraries were quantified using the Qubit dsDNA HS Assay Kit (Q32584; Invitrogen). Based on the results of Qubit quantitation, 10 ng of the library was taken for each sample and mixed in equal proportion.

The mixed libraries were again subjected to the Qubit quantitation assay. Then, single-end sequencing was performed on the HiSeq 2500 platform (Illumina, San Diego, CA, USA) under relevant parameters. The read length was set to 140 bp. The volume of raw data was approximately 5 M reads.

### Identification and functional annotation of the DEGs

The raw sequencing reads were filtered to exclude low-quality reads and alternative alignment with RNA-SeQC [41]. Qualified reads were mapped to the human reference genome (Ensembl primary assembly, version GRCh37) by using STAR [42]. The RNA expression level was estimated by FPKM [43] (fragments per kilobase million) of each gene. Base-2 logarithmic transformation of FPKM was conducted for further analyses.

DEGs among the different ER conditions were identified by analysis of variance (ANOVA) with the following equation: $Y_{gijk} = \mu_{g} + T_{gi} + S_{gj} + \varepsilon_{gijk}$, where $\mu_{g}$ represents the mean expression level of gene $g$; $T_{gi}$ is the gene-specific treatment effect referring to whether the patient had a natural cycle or was undergoing hormone replacement therapy when the endometrial tissue was obtained, $T_{gi} \sim (0,\sigma_{{T_{g} }}^{2} )$; $S_{gj}$ is the gene-specific ER stage effect with three levels (prereceptivity, receptivity, and postreceptivity), $S_{gi} \sim (0,\sigma_{{S_{g} }}^{2} )$; and $\varepsilon_{ijgk}$ is the gene-dependent residual error, $\varepsilon_{ijgk} \sim (0,\sigma_{{\varepsilon_{g} }}^{2} )$. The F-test was applied to statistically assess the equality of variances between $S_{j}$ and $\varepsilon_{ijk}$ for each gene, showing whether the gene is differentially expressed among the different ER stages. Because RNA-Seq analysis involves multiple statistical tests, the false discovery rate (FDR) was used to adjust the *P*-value (q-value) to provide statistical inference [44] . Functional analysis of these DEGs was conducted by the DAVID tool based on the Gene Ontology (GO) categories, namely, biological process, cellular component and molecular function, and Kyoto Encyclopedia of Genes and Genomes (KEGG) pathways.

### Candidate marker gene selection and predictive tool construction

The samples from the first phase were used as a training dataset for prediction model construction of ER status. The cutoff q-value of 1e−10 was used to select the DEGs. The expression values of these DEGs were then inputted as features for the random forest machine learning method to train the pattern on three ER conditions (prereceptivity, receptivity, and postreceptivity). The importance of each feature (gene expression) was calculated with the R package random Forest by mean decrease accuracy measure. The mean accuracy from tenfold cross-validation, and area under the receiver operating characteristic (ROC) curve (AUC) were calculated to evaluate the performance of the predictor.

### pET guided by the rsERT and outcome measures

In the first frozen ET cycle after rsERT in the experimental group, pET was performed at the timing of optimal WOI predicted by rsERT, which corresponds to the transfer of blastocysts, and day-3 cleavage-stage embryos were transferred 2 days earlier accordingly. Patients in the control group underwent conventional ET directly (i.e., transfers of frozen–thawed embryos or blastocysts were performed 5 or 7 days after the LH surge or 3 or 5 days after progesterone supplementation).

The primary outcome measure was the intrauterine pregnancy rate (IPR). Secondary outcomes were live birth rate (LBR) and implantation rate (IR). We have adopted the following standardized definitions [45]. *IPR* refers to the number of patients with intrauterine pregnancy per ET cycle. *LBR* refers to the number of deliveries that resulted in at least one live birth per ET cycle. *IR* refers to the number of gestational sacs observed divided by the number of embryos transferred, with a single ET gestation sac counting as 1 only.

### Statistical analysis

Continuous data subject to a normal distribution are expressed as the mean ± SD and were compared using independent-samples *t*-tests. Continuous data subject to a skewed distribution are expressed as the median and interquartile range (IQR) and were compared using the independent-samples Mann–Whitney *U* test. Categorical data are expressed as counts and percentages and were determined to be statistically significant using the chi-square test or Fisher’s exact test. A two-sided *P*-value equal to or less than 0.05 was considered to be statistically significant. Statistical analysis was performed using IBM SPSS software (Version 23.0, IBM Corp.).

## Results

### Participants

**Fig. 1:**
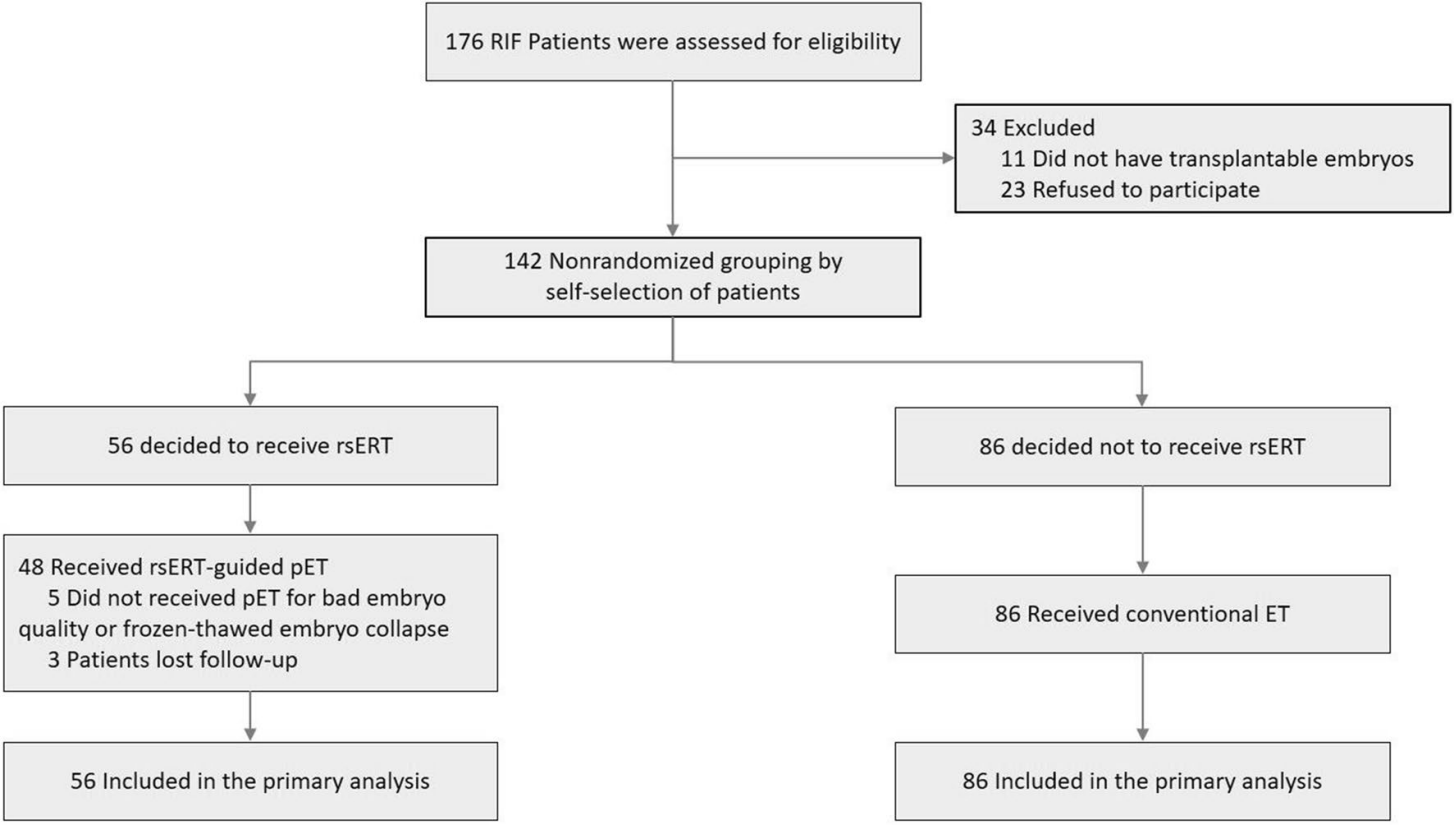
Flow chart of participants in the rsERT-guided pET trial (the second phase of the current study)

In the first phase, 71 participants were recruited, 21 patients who were not pregnant after the first ET were excluded, and 50 patients with successful intrauterine pregnancies were used to build the rsERT model. The baseline clinical characteristics are shown in Table 1. In the second phase, 56 of the 142 enrolled patients with RIF were assigned to the experimental group and 86 to the control group by self-selection. The baseline clinical characteristics were comparable among groups (Table 2). The percentage of blastocysts (day 5 or day 6) transferred in the experimental group was significantly different from that in the control group (*P* = 0.013), while there was no significant difference in the percentage of high-quality day 3 cleavage-stage embryos (44/51, 86.3% vs. 105/114, 92.1%, *P* = 0.376) and blastocysts (17/39, 43.6% vs. 19/44, 43.2%, *P* = 0.970) between the two groups. The two groups also showed no significant differences in the other variables. In the experimental group, 48 of 56 patients with RIF received rsERT-guided pET; however, for 5 patients had poor-quality or collapsed freeze–thaw embryos, and 3 patients were lost to follow-up. All 86 patients with RIF in the control group underwent conventional ET (Fig. 1).

**Table 1:** Demographic clinical characteristics of the IVF patients with rsERT

Characteristic	The first phase study (n = 50)
Age, mean ± SD, y	30.9 ± 3.89
BMI, mean ± SD, kg/m^2^	21.0 ± 2.19
Infertility duration, median (IQR), y	3 (1.0–5.5)
AMH, median (IQR), ng/ml	3.21 (2.39–5.33)
FSH, mean ± SD, mIU/ml	5.63 ± 1.15
AFC, median (IQR)	13 (9–15.75)
Endometrial thickness, mean ± SD, mm	11.0 ± 2.74
IVF indication	
Male factor (n)	6
Tubal factor (n)	44

*IVF* in vitro fertilization, *rsERT* RNA-Seq-based endometrial receptivity test, *BMI* body mass index, *AMH* anti-Mullerian hormone, *FSH* follicle-stimulating hormone, *AFC* antral follicle count

**Table 2:** Baseline clinical characteristics of the patients with RIF in the experimental and control groups

Characteristic	Experimental (n = 56)	Control (n = 86)	*P*-value
No. of previous failed cycles, median (IQR)	3 (2–4)	3 (3–4)	0.462
Age, mean ± SD, y	32.71 ± 4.14	32.90 ± 3.79	0.789
BMI, mean ± SD, kg/m^2^	21.38 ± 2.39	21.41 ± 1.85	0.926
Infertility duration, mean ± SD, y	5.18 ± 3.42	4.38 ± 3.29	0.168
AMH, median (IQR), ng/ml	2.86 (1.40–5.33)	4.10 (2.31–6.15)	0.154
FSH, mean ± SD, mIU/ml	6.49 ± 1.59	6.27 ± 1.67	0.478
AFC, mean ± SD	12.55 ± 6.91	13.92 ± 6.50	0.261
Endometrial thickness, mean ± SD, mm	9.47 ± 1.85	9.26 ± 1.42	0.469
P levels on the day of progesterone administration/LH peak, median (IQR), ng/ml	0.31 (0.09–0.61)	0.29 (0.15–0.72)	0.529
Types of infertility			
Primary infertility (n/%)	33 (58.9%)	41 (47.7%)	0.190
Secondary infertility (n/%)	23 (41.1%)	45 (52.3%)	
IVF indication			
Male factor (n/%)	2 (3.6%)	3 (3.5%)	0.704
Tubal factor (n/%)	50 (89.3%)	82 (95.3%)	
PCOS (n/%)	6 (10.7%)	11 (12.8%)	
Diminished ovarian reserve (n/%)	6 (10.7%)	9 (10.5%)	
Endometriosis (n/%)	5 (8.9%)	3 (3.5%)	
Others (n/%)	2 (3.6%)	1 (1.2%)	
Sampling cycle protocol			
Natural cycle (n/%)	26 (46.4%)	34 (39.5%)	0.416
HRT cycle (n/%)	30 (53.6%)	52 (60.5%)	
No. of transferred embryos, median (IQR)	2 (2–2)	2 (2–2)	0.608
Total no. of transferred embryos (n)	90	158	
Embryo stage			
D3 cleavage-stage embryos (n/%)	51 (56.7%)	114 (72.2%)	**0.013**
D5 or D6 blastocysts (n/%)	39 (43.3%)	44 (27.8%)	

Bold value indicates statistical significance

*BMI* body mass index, *AMH* anti-Mullerian hormone, *FSH* follicle-stimulating hormone, *AFC* antral follicle count, *P levels* serum endogenous progesterone level, *PCOS* polycystic ovarian syndrome, *HRT cycle* hormone replacement cycle

### Identification of DEGs among ER statuses

To identify DEGs among prereceptivity, receptivity, and postreceptivity stages that could then be used as biomarkers to predict ER, we compared the transcriptomes of endometrial tissues collected at LH + 5, LH + 7 and LH + 9 days for patients with a natural cycle. Briefly, we constructed 150 NGS libraries by using the total RNA extracted from endometrial biopsy samples of the 50 patients recruited in the first phase. An average of 6.7 M raw reads were generated from 146 qualified libraries, with the mapping rate ranging from 63.7 to 96.0%. Each library detected 14,507 genes on average, resulting in a total of 3571 DEGs within the three different ER statuses, representing approximately 17% of all mapped genes.

**Fig. 2:**
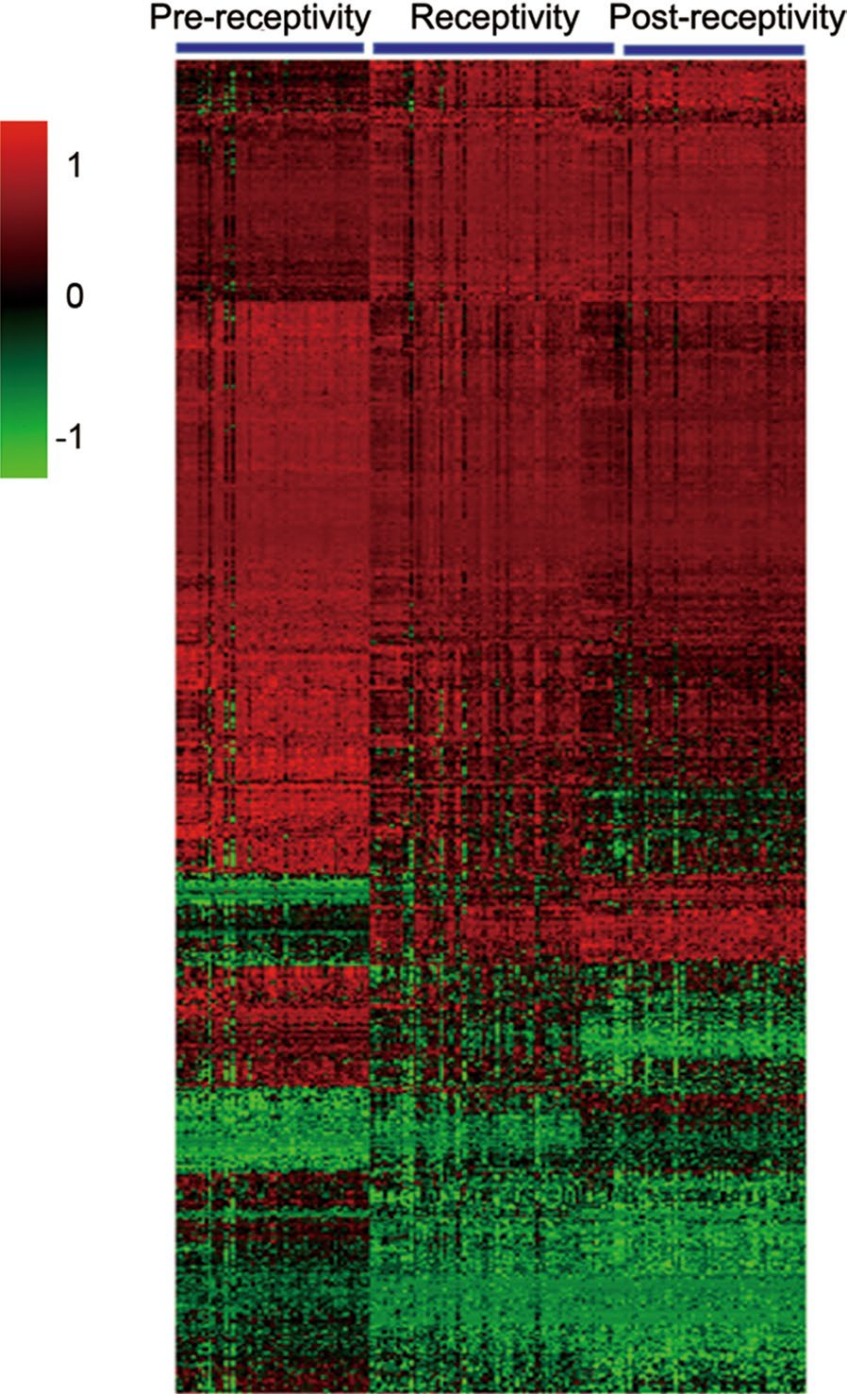
Hierarchical clustering of the RNA expression data from 50 individuals; three samples per individual were obtained, one at each ER stage

Three well-defined groups were generated by clustering analysis (Fig. 2), in agreement with the timing of sampling. Functional analysis showed significant enrichment in embryo-endometrium interaction and embryonic implantation-related processes, such as protein transport (GO: 001503), cell–cell adhesion (GO: 0098609) and the oxidation–reduction process (GO: 0055114) in the biological process category; protein binding (GO: 0005515) and protein homodimerization activity (GO: 0042803) in the molecular function category; and cell–cell adherens junction (GO: 0005913), extracellular exosome (GO: 0070062) and focal adhesion (GO: 0005925) in the cellular component category. KEGG pathways, including ECM-receptor interaction and signal transduction-related molecular function-like protein kinase binding, were also enriched among these DEGs (Additional file 1).

### Establishing and validating the ER predictive tool

**Fig. 3:**
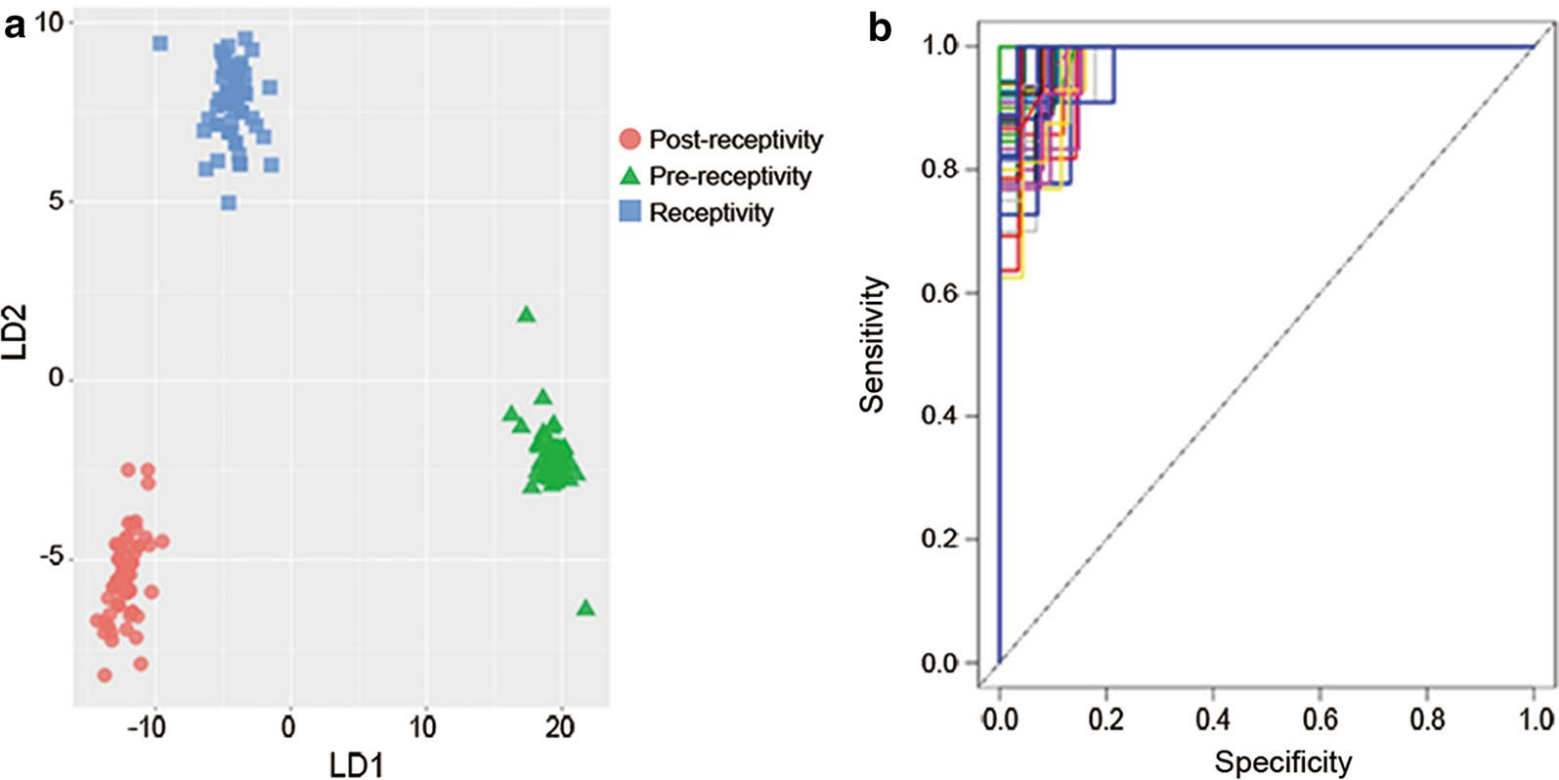
Establishment and validation of the RNA-Seq-based endometrial receptivity test (rsERT). **a** Linear discriminant analysis (LDA) of endometrial receptivity conditions based on selected predictive markers. **b** ROC curves generated by 100 random splits into a training set and a test set

Next, we used the DEGs to construct a predictive model for the three ER conditions. The random forest algorithm was applied to train the model to recognize the pattern of RNA expression, resulting in predictive markers containing 175 genes with mean decrease accuracy ranging from 3 to 5.43. Linear discriminant analysis (LDA) showed that the three ER conditions (prereceptivity, receptivity, and postreceptivity) were distinctly classified by the expression pattern of these predictive markers. (Fig. 3a and Additional file 2). The average of tenfold cross-validation was applied to assess the performance of the predictive model, resulting in a mean accuracy of 98.4% with 98.9% specificity and 97.8% sensitivity. ROC curve analysis of 100 random splits into a training set and a test set yielded an average area under the curve (AUC) of 99.1% (Fig. 3b).

### rsERT results in patients with RIF

In the second phase of the study, a total of 168 NGS libraries were constructed for RNA-Seq by using endometrial biopsy samples from patients in the experimental group (n = 56), with a qualification rate of 96.4% (162 of 168). The expression profile of selected markers was utilized to predict ER status. The results indicated WOI displacement in 17 of 56 patients (30.4%). Among them, advanced WOI occurred in 15 patients (15/17, 88.2%), and delayed WOI occurred in 2 patients (2/17, 11.8%). WOI displacement was combined with narrowing in 10 (10/17, 58.8%) patients (WOI < 48 h).

### Effect of rsERT-guided pET on pregnancy outcomes in RIF

Considering the significant difference in the percentage of transferred blastocysts between the experimental group and the control group, we compared the pregnancy outcomes of transferred day-3 cleavage-stage embryos and blastocysts separately. The results showed that 26 of 48 patients in the experimental group and 59 of 86 patients in the control group had transferred day-3 embryos. The IPR (13/26, 50%) and IR (16/51, 31.4%) of experimental group were significantly higher than the IPR (14/59, 23.7%) and IR (19/114, 16.7%) of the control group, respectively (RR, 2.107; 95% CI 1.159 to 3.830; *P* = 0.017, RR, 1.882; 95% CI 1.057 to 3.353; *P* = 0.033). The LBR (11/26, 42.3%) in the experimental group was 20% higher than that (13/59, 22%) in the control group, although the difference was not statistically significant (RR, 1.92; 95% CI 0.995 to 3.705; *P* = 0.056). In addition, 22 patients in the experimental group and 27 patients in the control group underwent blastocyst transplantation. The IPR, LBR and IR (63.6%, 59.1% and 43.6%) in the experimental group were all distinctly higher than those (40.7%, 37% and 27.3%) in the control group but not significantly different (RR, 1.562; 95% CI 0.898 to 2.718; *P* = 0.111, RR, 1.595; 95% CI 0.874 to 2.914; *P* = 0 0.124, RR, 1.598; 95% CI 0.877 to 2.913; *P* = 0 0.120) (Table 3).

**Table 3:** Pregnancy outcomes of pET in the experimental group and conventional ET in the control group

	Experimental			Control (n = 86)	*P*-value^a^	*P*-value^b^	*P*-value^c^	RR^e^ (95%Cl)	*P*-value^d^
	Displaced (n = 17)	Non-displaced (n = 39)	Total (n = 56)						
No. of patients receiving embryo transfers (n)	16	32	48	86					
No. of patients transferred D3 embryos (n)	8	18	26	59					
No. of patients who received pET (n)	8	18	26	–					
Intrauterine pregnancy (n/%)	5/8 (62.5%)	8/18 (44.4%)	13/26 (50%)	14/59 (23.7%)	0.062	0.089	0.673	**2.107 (1.159 to 3.830)**	**0.017**
Live birth (n/%)	4/8 (50%)	7/18 (38.9%)	11/26 (42.3%)	13/59 (22.0%)	0.203	0.263	0.683	1.92 (0.995 to 3.705)	0.056
No. of transferred D3 embryos (n)	15	36	51	114					
Embryos implanted (n/%)	6/15 (40%)	10/36 (27.8%)	16/51 (31.4%)	19/114 (16.7%)	0.072	0.141	0.599	**1.882 (1.057 to 3.353)**	**0.033**
No. of patients transferred blastocysts (n)	8	14	22	27					
No. of patients who received pET (n)	8	14	22	–					
Intrauterine pregnancy (n/%)	6/8 (75%)	8/14 (57.1%)	14/22 (63.6%)	11/27 (40.7%)	0.121	0.318	0.649	1.562 (0.898 to 2.718)	0.111
Live birth (n/%)	6/8 (75%)	7/14 (50%)	13/22 (59.1%)	10/27 (37.0%)	0.105	0.424	0.380	1.595 (0.874 to 2.914)	0.124
No. of transferred blastocysts (n)	13	26	39	44					
Embryos implanted (n/%)	7/13 (53.8%)	10/26 (38.5%)	17/39 (43.6%)	12/44 (27.3%)	0.147	0.330	0.497	1.598 (0.877 to 2.913)	0.120

Bold *P*-values indicates statistical significance

*RR* relative risk, *pET* personalized embryo transfer, *ET* embryo transfer

^a^Indicating the p-value of the displaced group compared with the control group

^b^Indicating the p-value of the non-displaced group compared with the control group

^c^Indicating the p-value of the displaced compared with the non-displaced group

^d^Indicating the p-value of the experimental group compared with the control group

^e^Indicating the RR of the experimental group compared with the control group

**Fig. 4:**
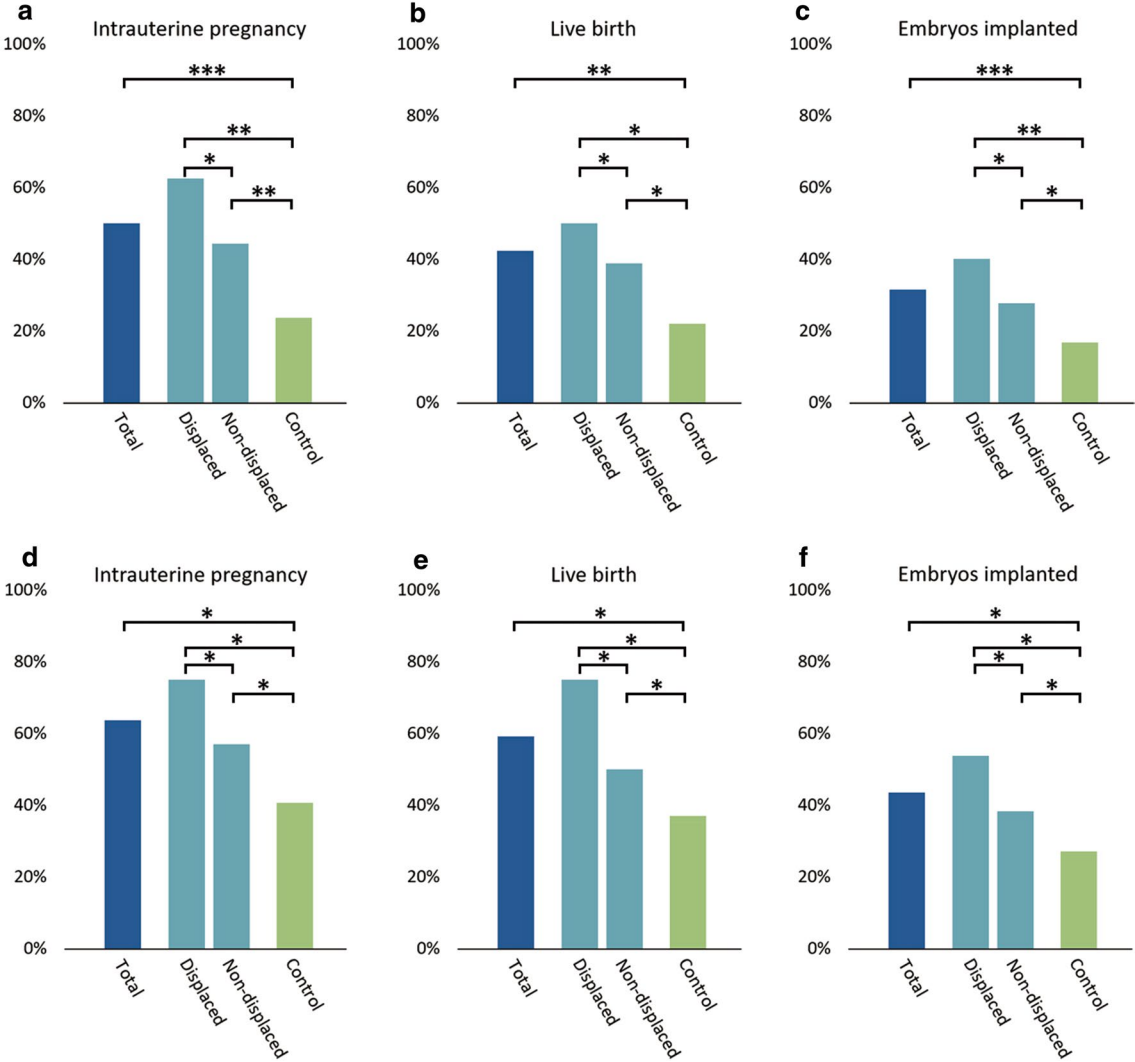
Comparison of pregnancy outcomes in experimental and control group. **a**–**c** IPR, LBR and IR for patients transferred day 3 embryos; **d**–**f** IPR, LBR and IR for patients transferred blastocysts

To determine whether the improvement of pregnancy outcomes in patients with RIF is attributed to the prediction of optimal WOI by rsERT, we analyzed separately pregnancy outcomes of patients with and without WOI displacement in the experimental group. 16 of 17 patients in the WOI displaced group underwent pET, including 8 with day 3 embryos and 8 with blastocysts. 32 of 39 patients in the WOI non-displaced group received pET, 18 with day 3 embryos and 14 with blastocysts. There was no significantly different in the percentage of high-quality day 3 cleavage-stage embryos (13/15, 86.7% vs. 31/36, 86.1% vs. 105/114, 92.1%, *P* = 0.522) and blastocysts (6/13, 46.2% vs. 11/26, 42.3% vs. 19/44, 43.2%, *P* = 0.974) among the displaced, non-displaced and control groups. Whether day 3 embryos or blastocysts were transferred, the IPR, LBR and IR in the WOI displaced group were significantly higher than those in the control group, although there was no statistical difference. The IPR, LBR and IR in the WOI non-displaced group were higher than those in the control group, but there was no significant difference. The IPR, LBR and IR in the WOI displaced group were higher than those in the non-displaced group and there was no significant difference. Detailed data are shown in Table 3 and Fig. 4.

## Discussion

RIF is a highly challenging condition in ART with a complex etiology. Embryo quality and maternal endometrial factors are the main causes of RIF. At present, increasing attention has been given to the effect of abnormal ER on RIF. WOI displacement that disrupts the synchronicity of embryonic and endometrial development is a crucial cause of RIF. Therefore, in the current study, we aimed to use RNA-Seq to identify biomarkers for ER through transcriptome analysis and create a novel rsERT tool to accurately predict the optimal WOI and to improve the pregnancy outcomes of patients with RIF by rsERT-guided pET.

Through RNA-Seq, over 3000 DEGs were identified in our study, and GO annotation and KEGG pathway analyses showed marked enrichment in embryo implantation process-related functions and pathways, such as cell–cell adhesion, focal adhesion, ECM-receptor interaction and signal transduction-related processes like FoxO signaling pathway. Interestingly, metabolic pathway were enriched in these DEGs, implying its role of supplying endometrial requirements like energy or structural composition for endometrium-embryo talk.

To date, several studies have revealed transcriptome changes during different receptivity stages, indicating the reliability and universality of DEGs as predictive biomarkers for ER [46–48]. However, unlike previous studies, we screened DEGs based on the following experimental methods and design advantages. First, RNA-Seq used for sequencing analysis can provide a more precise and comprehensive view of transcriptome changes in the endometrial cycle than the conventional gene microarray. Second, comparing the sequencing data of the endometrial tissue samples (LH + 5/LH + 7/LH + 9) among three different receptive states collected from the same patient at 48-h intervals during the same cycle can allow a more precisely analysis of the DEGs to identify marker genes for ER due to narrow comparison time span. Third, the receptive period used as the contrast point was defined as day LH + 7 determined by combining the blood LH surge with a subsequent intrauterine pregnancy, which is more reliable than the previous determination with the LH surge alone, so that the obtained DEGs are more accurate. Finally, for the first time, we collected largest sample size of endometrial tissue from the Chinese population to build the ER prediction model, which should be more suitable for the detection of the Chinese and Asian populations than previous prediction methods applied in Europe and America. As a result, 175 markers were selected from DEGs, and the rsERT was established by applying the random forest algorithm. The accuracy, specificity and sensitivity of the rsERT for predicting the optimal WOI by tenfold cross-validation were 98.4%, 98.9% and 97.8%, respectively.

In the second phase study, rsERT was applied for patients with RIF, resulting in a WOI displacement rate of 30.4%, which was similar to that in other studies [49, 50]. However, unlike previous studies, most WOI displacement was advanced rather than delayed, which may be related to race, the small sample size and regional population, and more clinical verification is needed. In addition to WOI displacement, 58.8% of patients with RIF also exhibited narrowing of the WOI, which allows a shorter duration of WOI for embryo implantation, so it is particularly important to accurately predict the optimal WOI.

Subsequently, rsERT-guided pET was performed for patients in the experimental group. Compared with those in the control group, the IPR, LBR and IR were higher in the experimental group, especially for patients who had transfers of day-3 cleavage-stage embryos. The differences in IPR and IR were statistically significant, while LBR was 20 percentage points higher than that in the control group (42.3% vs. 22%), though without statistical significance. IPR (63.6% vs. 40.7%), LBR (59.1% vs. 37%) and IR (43.6% vs. 27.3%) were not significantly different between the two groups when blastocysts were transferred. However, the IPR and LBR in the experimental group were higher than those in the control group by more than 20 percentage points, and the IR was also higher by 16 percentage points. In addition, our subgroup analysis also showed that the indicators of pregnancy outcome in the WOI displaced patients of the experimental group increased by more than 20 to 30 percentage points compared with the control group, regardless of the day 3 embryos or blastocysts transferred. This improvement was due to the restoration of the synchronicity of embryonic and endometrial development, rather than the influence of embryonic factors, as shown by the lack of significant differences in the proportion of good-quality day-3 cleavage-stage embryos and good-quality blastocysts transferred between groups. Our results also showed that IPR, LBR and IR were not significantly different in the non-displaced group compared to the control group, but were all increased by more than 10 percent points. This is because there were some patients with WOI displacement in the control group, and their embryos were transferred in the endometrial non-receptive period, which resulted in the worse pregnancy outcomes in this group. After personalized embryo transfer, the pregnancy outcomes in the displaced group was better than that in the non-displaced group, indicating that WOI displacement was the main cause of implantation failure in the displaced group and that pregnancy outcomes were effectively improved by adjusting the correct embryo transfer time. These results demonstrate that the use of pET guided by rsERT significantly improved the pregnancy outcomes of patients with RIF, especially those with WOI displacement.

After completing the construction and clinical validation of the rsERT model, another important objective of our study was to further optimize the detection model so that the optimal WOI period can be accurately predicted by one-point sampling. In fact, in our study, the DEGs changing patterns during the three periods of prereceptivity, receptivity and postreceptivity at 48-h intervals provided a good basis for data analysis to optimize the model for one-point sampling prediction. Therefore, based on our three-point sampling prediction results and corresponding clinical outcomes, an optimal WOI point estimation method with hour precision by one-point sampling was developed and is being clinically validated.

Nonetheless, this study had several limitations. First, the sample size of this study was small, and there were no application data for the rsERT in infertile patients with conventional IVF. To clarify the clinical value of rsERT in infertile populations with conventional IVF, we think it would be better to design a multicenter randomized controlled trial of rsERT combined with PGT in the future. Second, according to the results of this study, 43.7% of patients with RIF still experienced implantation failure after pET guided by rsERT. Therefore, it can be inferred that in these patients, in addition to WOI displacement, there may be ER pathological disruption. Another limitation of this study is that we cannot diagnose the pathological disruption of ER by evaluating the strength of WOI receptivity capacity. Our future work will identify marker genes representing WOI receptive capacity to establish a new ER detection model to diagnose pathological disruption of ER and to study the mechanism of ER marker genes to provide a theoretical basis for clinical treatment strategies. Of course, performing PGT to exclude embryonic factors is also an option to consider. Third, because of the invasive operation of endometrial sampling, noninvasive ERT is also our future research direction.

## Conclusions

In summary, we built a novel rsERT that accurately predicts the WOI period. It consists of ER-specific marker genes and is screened by the combination of RNA-Seq and machine learning. rsERT-guided pET significantly improved the pregnancy outcomes of patients with RIF, indicating the clinical potential of rsERT-guided pET.

## Supplementary Information

**Additional file 1.** GO and KEGG annotation.

**Additional file 2.** List of predictive markers.

## Data Availability

The primary data for this study are available from the corresponding author upon reasonable request.

## Abbreviations

ER: Endometrial receptivity
WOI: Window of implantation
RNA-Seq: RNA sequencing
rsERT: RNA-Seq-based endometrial receptivity test
ET: Embryo transfer
pET: Personalized embryo transfer
RIF: Repeated implantation failure
ART: Assisted reproductive technology
IVF: In vitro fertilization
IVF-ET: In vitro fertilization and embryo transfer
DEGs: Differentially expressed genes
BMI: Body mass index
ERA: Endometrial receptivity array
FSH: Follicle-stimulating hormone
LH: Luteinizing hormone
HCG: Human chorionic gonadotropin
